# Extract of *Bacopa procumbens* Mitigates Brain Injury and Improves Functional Outcomes Following Ischemia–Reperfusion

**DOI:** 10.3390/ijms262411781

**Published:** 2025-12-05

**Authors:** José Manuel Viveros-Bartolomé, Salvador Pérez-Mora, Iván Alquisiras-Burgos, Ari Misael Martínez-Torres, Maribel Pérez-Rodríguez, Juan Ocampo-López, Yazmin Montserrat Flores-Martinez, María del Consuelo Gómez-García, Penélope Aguilera, David Guillermo Pérez-Ishiwara

**Affiliations:** 1 Laboratory of Molecular Biomedicine I, Programa de Doctorado en Ciencias en Biotecnología y Maestría en Biomedicina Molecular, Escuela Nacional de Medicina y Homeopatía (ENMyH), Instituto Politécnico Nacional, Mexico City 07320, Mexico; jviverosb2000@alumno.ipn.mx (J.M.V.-B.); sperezm1510@alumno.ipn.mx (S.P.-M.); aricoug5@gmail.com (A.M.M.-T.); mperezr2103@alumno.ipn.mx (M.P.-R.); yfloresma@ipn.mx (Y.M.F.-M.); cgomezg@ipn.mx (M.d.C.G.-G.); 2 Research Department, Atria Scientific, Av. de los Maestros 452, Nueva Santa María, Azcapotzalco, Mexico City 02800, Mexico; 3 Laboratory of Cerebral Vascular Pathology, Instituto Nacional de Neurología y Neurocirugía Manuel Velasco Suárez, Insurgentes Sur #3877, Mexico City 14269, Mexico; ialquisiras@innn.edu.mx (I.A.-B.); penelope.aguilera@innn.edu.mx (P.A.); 4 Laboratory of Histology and Histopathology, Academic Area of Veterinary Medicine, ICAp, Universidad Autónoma del Estado de Hidalgo, Tulancingo de Bravo, Hidalgo 43600, Mexico; jocampo@uaeh.edu.mx

**Keywords:** ischemic stroke, *Bacopa procumbens*, neuroprotective, antioxidant, lipid peroxidation, edema, activity prediction, interactome analysis

## Abstract

Ischemic stroke remains one of the leading causes of disability and mortality worldwide, and effective therapeutic options are still limited. Therefore, this study aimed to evaluate the neuroprotective effect of the aqueous extract of *Bacopa procumbens* (*B. procumbens*) in a murine model of ischemia/reperfusion induced by middle cerebral artery occlusion (MCAO). This widely used model is generated by the transient intraluminal insertion of a nylon filament through the external carotid artery to occlude the middle cerebral artery, allowing controlled induction and subsequent reperfusion. Wistar rats underwent 2 h MCAO, followed by tail vein administration of *B. procumbens* extract (40 mg/kg) or Edaravone (0.45 mg/kg) before reperfusion. Neurological, histological, and molecular parameters were assessed 48 h later. Additionally, in silico analyses were performed to predict the antioxidant activity of the extract’s major metabolites and to explore Nrf2-related signaling. *B. procumbens* treatment improved neurological condition, reduced the volume of the infarct lesion, increased the expression and activation of Akt and Nrf2, reduced lipid peroxidation (4-HNE), and downregulated AQP4, the main water channel involved in cerebral edema formation. These molecular effects were associated with enhanced neuronal survival and collectively resulted in significant neuroprotection in the MCAO model. In silico analysis identified key metabolites with high antioxidant potential through free radical scavenging, lipid peroxidation inhibition, and redox enzyme modulation. Nrf2-centered interactome analysis revealed eighty-two proteins linked to ischemia, neuroinflammation, neuronal death regulation, and oxidative stress response. These findings support the therapeutic potential of *B. procumbens* metabolites as neuroprotective agents against ischemic cerebral injury.

## 1. Introduction

Ischemic stroke (IS) is the second leading cause of death and the primary cause of long-term disability worldwide [1]. It is classified as either hemorrhagic or ischemic, with the latter being the most common, accounting for approximately 80% of all cases [2]. IS occurs when blood flow to a region of the brain is compromised, leading to cell death due to insufficient oxygen and nutrient supply [3]. The main causes of blood flow obstruction are clots or emboli and include cerebral vessel atherosclerosis and the rupture of atherosclerotic plaques [4,5].

The ischemic event triggers a series of cellular processes known as the ischemic cascade, in which oxidative stress, excitotoxicity, and inflammation contribute to neuronal damage [6,7]. First, cerebral ischemia causes a severe ionic imbalance, where anoxic depolarization induces Ca^2+^ overload due to excessive glutamate release, thereby amplifying excitotoxicity and oxidative stress [8]. Subsequently, mitochondrial damage increases the production of reactive oxygen species (ROS), compromising cell viability and activating cell death pathways via necrosis or apoptosis [9]. Additionally, cerebral edema, one of the major consequences of ischemia, beginning with cytotoxic edema caused by the intracellular accumulation of Na^+^ and osmolytes [10,11], which is exacerbated by the overexpression of aquaporin-4 (AQP4), the main water channel in the central nervous system [12,13]. Vasogenic edema later develops, compromising the blood–brain barrier and further aggravating tissue injury [14,15,16]. Finally, inflammation begins locally within minutes through the activation of resident immune cells such as microglia and astrocytes, which release pro-inflammatory cytokines, chemokines, and ROS. These mediators play a pivotal role in exacerbating oxidative stress, excitotoxicity, and edema, while also clearing cellular debris and promoting tissue repair [17,18].

Currently, the only FDA-approved treatment for ischemic stroke is recombinant tissue plasminogen activator (tPA). However, its effectiveness is limited to a therapeutic window of 4.5 h, and its administration carries a high risk of hemorrhage, making only about 10% of patients eligible for its use [19,20,21].

In Japan, Edaravone has been approved as an alternative therapy that can be administered within the first 24 h; however, prolonged use has been associated with acute renal failure [22,23]. Given these limitations, there is a growing interest in the research of natural compounds with neuroprotective potential, with the aim of expanding therapeutic alternatives.

Various plant extracts have shown beneficial effects in ischemia/reperfusion models [24,25]. Among these, *B. procumbens* has been identified as a plant of interest due to its high phenolic and polyphenolic content [26,27,28]. In addition to its applications in tissue regeneration [26,29,30,31], several studies have reported that some metabolites contained in the aqueous extract of *B. procumbens* possess neuroprotective properties against excitotoxicity, oxidative stress, and inflammation [32,33,34,35,36,37,38,39]. These findings suggest that *B. procumbens*, a Mexican endemic species, could represent a promising alternative for the treatment of ischemic stroke.

Despite the growing interest in plant-based therapies for brain protection, most studied extracts have been evaluated only for one or two isolated mechanisms—mainly oxidative stress or inflammation—without considering equally critical processes such as edema formation, neuronal survival, and the activation of Nrf2-dependent antioxidant pathways. In contrast, the aqueous extract of *B. procumbens* exhibits a distinctive phytochemical profile enriched in hydrophilic metabolites such as naringenin, equol 7-O-glucuronide, and paeoniflorin [32,38,40], whose combination and relative abundance are not observed in other traditional extracts used in ischemia models.

In this context, the middle cerebral artery occlusion (MCAO) model—one of the most widely used and well-established preclinical paradigms of focal cerebral ischemia—provides an ideal experimental framework to assess whether *B. procumbens* extract confers comprehensive neuroprotection by modulating multiple checkpoints of the ischemic cascade.

Therefore, this study aimed to determine the neuroprotective effects of the extract of *B. procumbens* in the ischemia/reperfusion injury induced by MCAO in Wistar rats. Our findings demonstrate that *B. procumbens* metabolites exerts significant neuroprotective activity in cerebral ischemia, as evidenced by improvements in neurological function, reductions in infarct volume, modulation of key molecular markers, and complementary in silico analyses supporting its antioxidant potential.

## 2. Results

### 2.1. B. procumbens Enhances Neurological Recovery After MCAO

**Figure 1:**
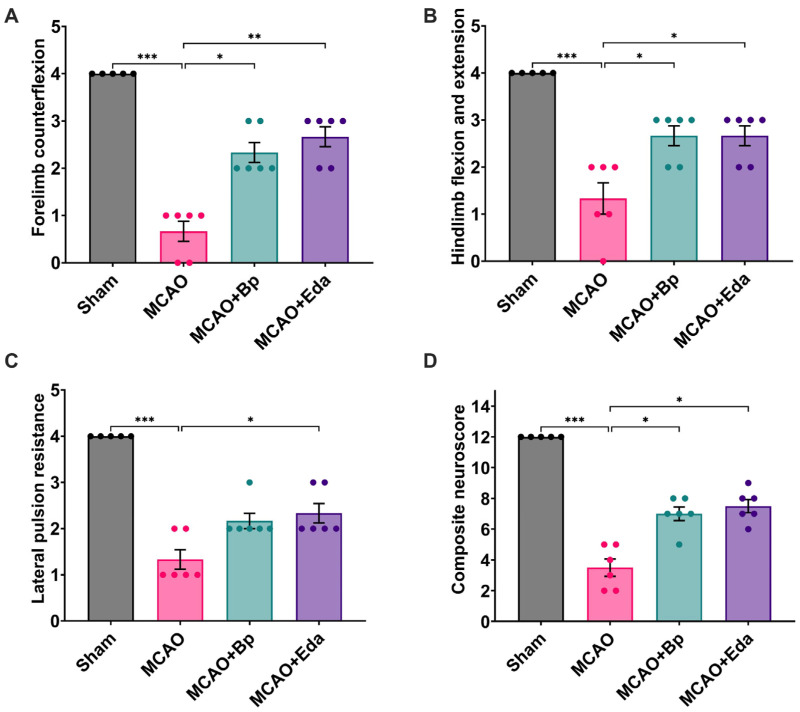
Effects of treatments on motor performance. (**A**) Forelimb retraction test; (**B**) Hindlimb flexion–extension test; (**C**) Lateral push resistance test; and (**D**) Composite neuroscore, calculated by summing the scores obtained in the three tests for each animal (maximum score: 12 points). All data are presented as mean ± SEM. Significant differences are indicated as (*), * *p* ≤ 0.05, ** *p* ≤ 0.01, and *** *p* ≤ 0.001. *n* = 6 per group.

A total mortality rate of 3.3% was observed among the ischemia-induced groups (1 out of 30 rats), with no deaths reported in the Sham group. Neurological deficits were evaluated in all experimental groups 48 h after ischemia/reperfusion injury. The results of each behavioral test were plotted individually (Figure 1A,C), and the total scores per animal were summed to obtain a composite neuroscore (Figure 1D).

The Sham group exhibited the highest scores across all behavioral assessments, reflecting normal neuromotor function. In contrast, the MCAO group displayed the lowest performance, with a maximum score of only 2 points, consistent with marked neurological impairment. Both MCAO + Bp and MCAO + Eda groups demonstrated significant improvements in all behavioral tests compared with the MCAO group. However, no statistically significant differences were found between the MCAO and MCAO + Bp groups in the lateral pulsion resistance test.

### 2.2. B. procumbens Reduces Infarct Volume and Decreases Histological Changes After Ischemia/Reperfusion Injury

**Figure 2:**
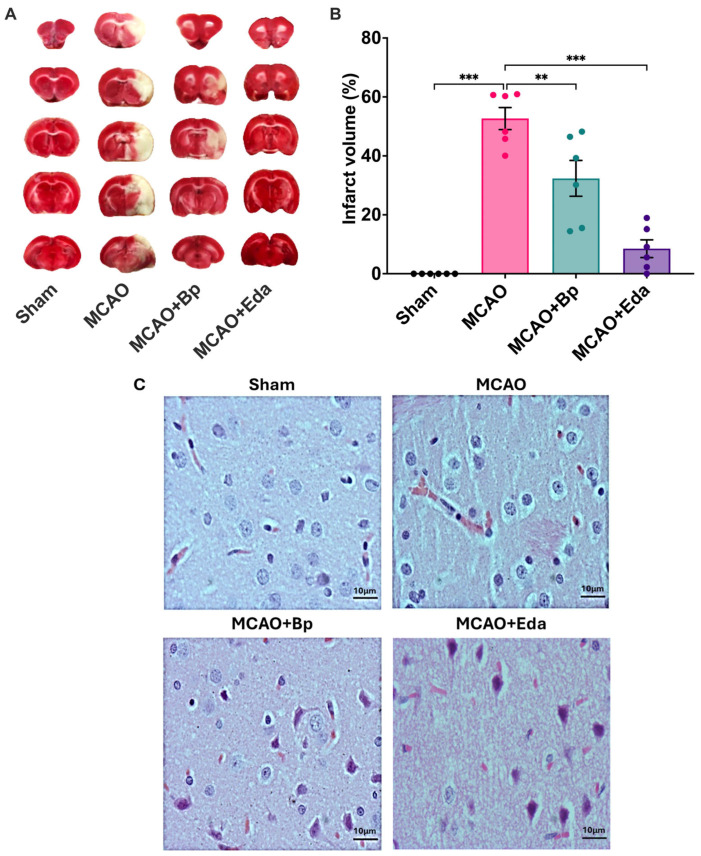
Neuroprotective effect of *B. procumbens* on brain damage after ischemia/reperfusion. (**A**) Representative TTC-stained coronal sections from each experimental group. Metabolically active tissue appears red, whereas pale or unstained regions correspond to infarcted areas. (**B**) Quantification of infarct volume expressed as the percentage of the affected area relative to the left hemisphere. (**C**) Histological evaluation of the cerebral cortex stained with H&E. Black arrows indicate pericellular spaces associated with edema. Scale bar: 10 µm. All data are presented as mean ± SEM. Statistical significance is denoted as (*), ** *p* ≤ 0.01, and *** *p* ≤ 0.001. *n* = 6 per group.

The lesion volume was quantified using 2,3,5-triphenyltetrazolium chloride (TTC) staining of coronal brain sections (Figure 2A,B). In the Sham group, no damage was observed, as the brain tissue stained entirely red, indicating cell viability. In contrast, in the MCAO group, the infarcted area accounted for nearly 52.66% of the total volume of the left hemisphere, as evidenced by the white regions in the brain slices. In the MCAO + Bp group, administration of *B. procumbens* extract significantly reduced the lesion volume, decreasing it to 32.3% of the affected hemisphere. Notably, Edaravone treatment further reduced the infarcted volume to approximately 8.5% of the left hemisphere. Unlike the results obtained in the neuroscore, TTC quantification showed a statistically significant difference between the Edaravone-treated group and the *B. procumbens*–treated group.

Histological analysis of cerebral cortical tissue stained with hematoxylin and eosin (H&E) revealed marked differences among the experimental groups (Figure 2C). In healthy animals, neuronal architecture remained intact, with no evidence of cytoplasmic retraction or pericellular spaces. In contrast, animals subjected to ischemia/reperfusion without treatment exhibited clear signs of tissue injury, including neuronal shrinkage, structural disorganization, and prominent perivascular edema—indicators of severe damage associated with the ischemic event. Treatment with *B. procumbens* markedly attenuated these alterations, showing better preservation of cellular morphology, fewer altered neurons, and a visible reduction in edema. Similarly, the Edaravone-treated group also displayed reduced neuronal damage, although perivascular edema persisted in some areas.

### 2.3. B. procumbens Reduces Lipid Peroxidation and Activates Nrf2 Protein

**Figure 3:**
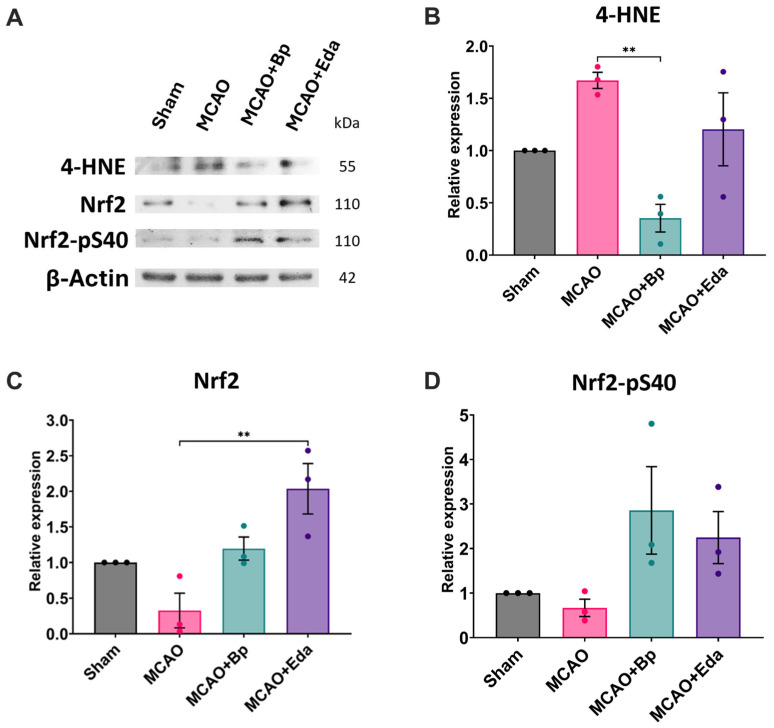
Evaluation of lipid peroxidation and activation of the endogenous antioxidant system. (**A**) Representative Western blot images for 4-HNE, total Nrf2, and Nrf2-pS40. (**B**) Quantification of 4-HNE, (**C**) total Nrf2, and (**D**) Nrf2-pS40. Relative protein expression levels were normalized to β-actin, using the Sham group as the baseline reference. Each biological replicate (*n* = 3 per group) corresponds to a pooled sample obtained by combining tissue from three rats. Data are presented as mean ± SEM. Significant differences are indicated as (*), ** *p* ≤ 0.01.

To assess the impact of *B. procumbens* on lipid peroxidation and the activation of the endogenous antioxidant system, 4-HNE levels and Nrf2 protein expression were analyzed. The MCAO group exhibited a 1.67-fold increase in 4-HNE relative to the Sham group, indicating heightened lipid peroxidation. In contrast, ischemic animals treated with *B. procumbens* showed a marked reduction in 4-HNE levels to 0.35-fold, surpassing the effect of Edaravone, which did not reduce lipid peroxidation below baseline values (Figure 3A,B).

Total Nrf2 expression decreased to 0.32-fold in the MCAO group compared with the Sham group, reflecting an impaired antioxidant response to ischemia/reperfusion injury. Treatment with *B. procumbens* or Edaravone restored Nrf2 expression to basal levels and further increased it to 1.19-fold and 2.03-fold, respectively (Figure 3A,C). Similarly, phosphorylated Nrf2 (Nrf2-pS40), the active form of the transcription factor, was reduced to 0.66-fold in the MCAO group relative to Sham. Conversely, administration of *B. procumbens* or Edaravone significantly elevated Nrf2-pS40 levels, reaching 2.85-fold and 2.24-fold increases, respectively (Figure 3A,D).

### 2.4. B. procumbens Regulates the Expression of Proteins Associated with Brain Edema and Neuronal Survival After Ischemia/Reperfusion

**Figure 4:**
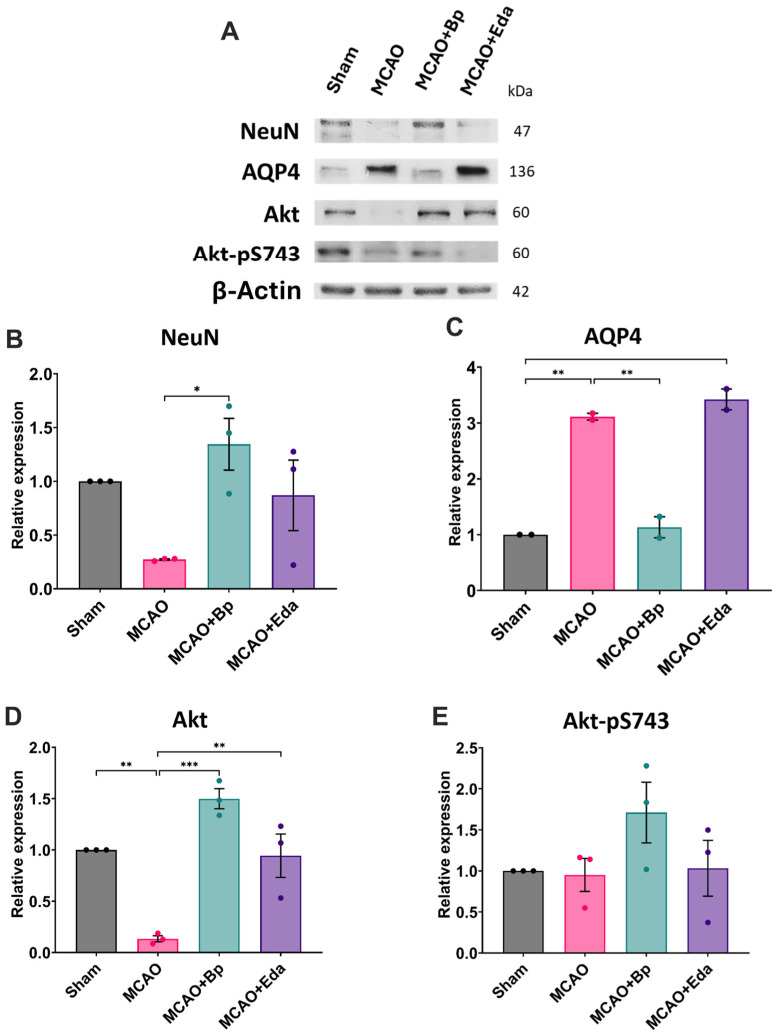
Evaluation of neuronal survival and anti-edematous effects of *B. procumbens* following ischemia/reperfusion injury. (**A**) Representative Western blot images showing the relative expression of NeuN, AQP4, total Akt, and Akt-pS473. (**B**) Quantification of NeuN, (**C**) AQP4, (**D**) total Akt, and (**E**) Akt-pS473 levels. Relative protein expression was normalized to β-actin, using the Sham group as the baseline reference. Each biological replicate (*n* = 3 per group) corresponds to a pooled sample obtained by combining tissue from three rats. Data are presented as mean ± SEM. Significant differences are indicated as (*), * *p* ≤ 0.05, ** *p* ≤ 0.01, and *** *p* ≤ 0.001.

To evaluate neuronal survival, the relative expression of NeuN was quantified. In the Sham group, NeuN levels reflected basal expression. In contrast, the MCAO group showed a marked reduction in NeuN, decreasing to 0.27-fold relative to the Sham group. Treatment with *B. procumbens* or Edaravone restored NeuN expression to 1.34-fold and 0.86-fold, respectively, reaching values comparable to those of the Sham group without statistically significant differences (Figure 4A,B).

Additionally, the MCAO group exhibited a 3.11-fold increase in AQP4 expression compared with the Sham group, indicating increased susceptibility to cerebral edema due to disrupted water homeostasis. Administration of *B. procumbens* reduced AQP4 expression to 1.13-fold relative to Sham, whereas Edaravone did not significantly modulate AQP4 levels (Figure 4A,C).

Given that Akt is a key regulator of cell survival pathways, both total Akt and its phosphorylated form (Akt-pS473) were evaluated. Total Akt expression was markedly reduced to 0.13-fold of the Sham value in the MCAO group. Conversely, treatment with *B. procumbens* significantly increased total Akt levels to 1.49-fold relative to Sham, while Edaravone produced a value of 0.94-fold (Figure 4A,E). Regarding Akt-pS473, similar levels were observed in the Sham, MCAO, and MCAO + Eda groups (0.95-fold and 1.03-fold relative to Sham, respectively). In contrast, *B. procumbens* treatment significantly increased Akt-pS473 expression, reaching 1.71-fold compared with the Sham group (Figure 4A,D).

### 2.5. Prediction of Antioxidant Activities

**Figure 5:**
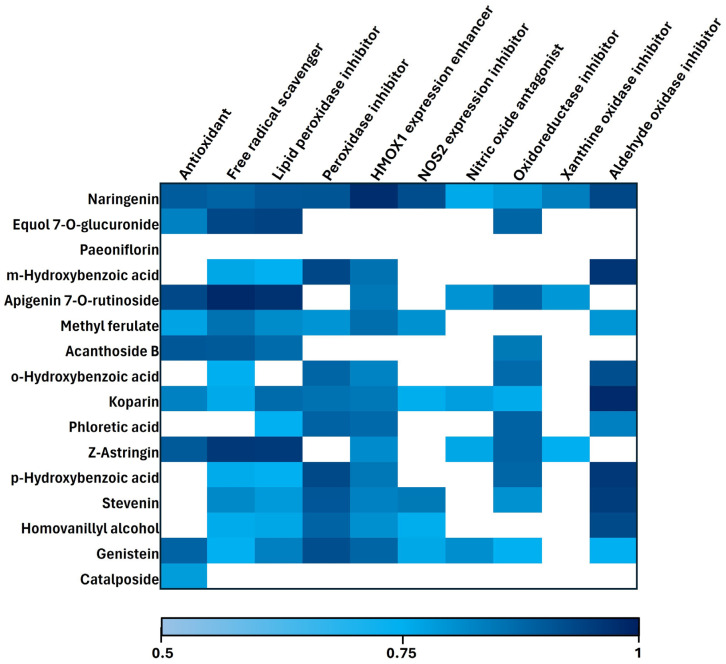
In silico antioxidant activity profile of *B. procumbens* metabolites. The blue gradient represents the predicted probability of each metabolite exhibiting a specific activity, according to PASS Online analysis. Blank cells indicate activities with a predicted probability below 0.5 or the absence of predicted activity for the corresponding compound.

To identify which *B. procumbens* metabolites may contribute to the antioxidant effects observed in our murine model of cerebral ischemia/reperfusion, predictive analyses were performed using the PASS Online platform. The results indicated that several metabolites present in *B. procumbens* display a potentially robust antioxidant functional profile. These activities were classified into three major mechanisms of action: (i) free radical scavenging, (ii) inhibition of lipid peroxidation, and (iii) modulation of key enzymes involved in oxidative metabolism (Figure 5).

A notable observation was the predicted synergistic action of several metabolites—including Naringenin, Equol 7-O-glucuronide, Apigenin 7-O-rutinoside, Acanthoside B, Koparin, Z-Astringin, and Genistein—each exhibiting high activity against molecular targets associated with oxidative stress. These included antioxidant capacity, free radical scavenging potential, and inhibition of lipid peroxidation. Additionally, these metabolites showed predicted affinity for modulating enzymes involved in oxidative metabolism, such as peroxidases, oxidoreductases, xanthine oxidase, and aldehyde oxidase, further supporting their multifunctional antioxidant profiles.

Among the analyzed compounds, Naringenin demonstrated the broadest and most potent bioactive profile, with high predicted probabilities for antioxidant activity (0.79), free radical scavenging (0.77), inhibition of lipid peroxidation (0.82), and peroxidase inhibition (0.82). It also exhibited strong predicted ability to induce HMOX1 expression (0.96), inhibit NOS2 expression (0.85), act as a nitric oxide antagonist (0.52), and inhibit oxidoreductases (0.58) and aldehyde oxidase (0.87).

Apigenin 7-O-rutinoside also presented elevated predicted activity as a free radical scavenger (0.97), lipid peroxidation inhibitor (0.94), and antioxidant (0.86), along with potential to induce HMOX1 (0.70). Similarly, Z-Astringin showed high activity in the same functional categories and inhibited xanthine oxidase (0.51).

Genistein displayed a particularly strong predicted profile as an antioxidant (0.77), peroxidase inhibitor (0.84), HMOX1 inducer (0.76), and lipid peroxidation inhibitor (0.67). To a lesser extent, it exhibited activity as a free radical scavenger (0.50), nitric oxide antagonist (0.62), and inhibitor of oxidoreductases (0.50) and aldehyde oxidase (0.50).

Other compounds, such as *m*-hydroxybenzoic acid, *p*-hydroxybenzoic acid, and Koparin, demonstrated strong predicted inhibitory activity against aldehyde oxidase (0.93, 0.92, and 0.97, respectively).

In contrast, Paeoniflorin did not display relevant predicted activity in the evaluated functions, potentially due to low structural representation in the training dataset or inherent limitations of the predictive approach. As a comparative control, the antioxidant Edaravone was also included; however, it exhibited probabilities below 0.5 in all predicted activities. Due to its consistently low predictive profile, its results were not included in the figure.

### 2.6. Interactome and Functional Enrichment Analysis of Nrf2 Focused on Its Antioxidant Role

To predict Nrf2-mediated regulation in our murine model and explore additional molecular pathways potentially modulated during ischemia/reperfusion, an interactome and functional enrichment analysis centered on this protein was performed using the *Rattus norvegicus* proteome as reference. The analysis identified a total of 82 proteins functionally associated with Nrf2, all with interaction probabilities ≥ 0.7, indicating a highly reliable and biologically meaningful network.

**Figure 6:**
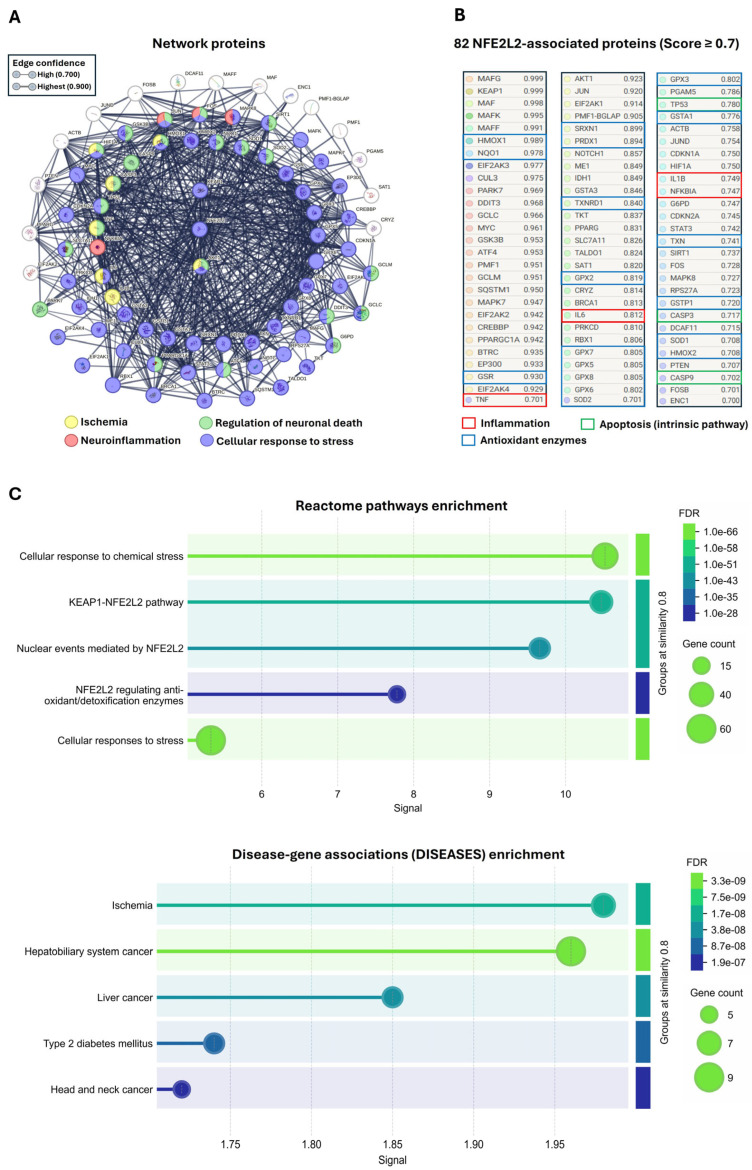
Interactome and functional enrichment analysis of Nrf2 (NFE2L2). (**A**) Nrf2 interactome network generated using the *Rattus norvegicus* proteome (interaction score ≥ 0.7). Proteins associated with ischemia (yellow), neuroinflammation (red), neuronal death regulation (green), and cellular stress response (blue) are highlighted. (**B**) List of the 82 proteins functionally associated with Nrf2, with selected proteins highlighted according to functional category: inflammation (red), apoptosis (green), and antioxidant enzymes (blue). (**C**) Functional enrichment analysis performed using REACTOME and DISEASES. The false discovery rate (FDR) is represented using a color scale (greener shades indicate higher statistical significance), while circle size denotes the number of genes associated with each pathway. Higher values along the X-axis indicate a greater enrichment score, reflecting pathways that are significantly overrepresented relative to random expectation.

Among the key components of this network are KEAP1, the canonical negative regulator of Nrf2, and Akt, an indirect activator that interacts with KEAP1 to facilitate Nrf2 release and subsequent activation. The interactome also includes proteins implicated in ischemic injury, such as IL-1β, IL-6, TNF-α, CASP3, HMOX1, and HIF-1. Additionally, several proteins involved in regulating neuronal death were identified, including PARK7, TNF-α, JUN, FOXO, and SIRT1. In the context of neuroinflammation, Nrf2 interactions were observed with NFKB, JUN, FOXO, and MAPK8. Apoptosis-related proteins such as p53, caspase-3, and caspase-9 were also highlighted, along with major stress-response elements including HMOX1, HMOX2, NQO1, SOD1, SOD2, GSR, TXN, and multiple glutathione peroxidases (GPXs) (Figure 6A,B).

Furthermore, functional enrichment analysis using REACTOME revealed that the Nrf2-associated interactome is primarily engaged in cellular responses to oxidative and chemical stress. Key enriched pathways included modulation of the Nrf2/KEAP1 signaling axis, Nrf2-mediated nuclear events, and transcriptional regulation of antioxidant and detoxification enzymes. Notably, disease-based enrichment analysis showed a strong association with ischemia, followed by several types of cancer and type 2 diabetes (Figure 6C).

## 3. Discussion

Overall, our findings indicate that the aqueous extract of *B. procumbens* confers neuroprotection in the MCAO model through the coordinated modulation of antioxidant defenses, edema regulation, and signaling pathways associated with neuronal survival. This integrated response suggests that the extract acts as a broad modulator of ischemic injury rather than functioning exclusively as an antioxidant.

From a functional standpoint, both *B. procumbens* and Edaravone improved neuro-motor performance at 48 h; however, the extent of motor recovery did not fully align with the differences observed in tissue preservation between treatments. This divergence has been reported in other MCAO studies and is likely attributable to the topographical distribution of ischemic damage rather than infarct size alone. Indeed, the localization of lesions, such as hemisphere asymmetry, has a strong influence on functional outcome [41]; detailed mapping in rodent models shows that perilesional and even remote regions may be affected beyond the infarct core [42]. Moreover, inter-strain differences in lesional evolution also point to variable spatial dynamics in ischemic damage that may decouple infarct volume from behavioral recovery [43]. In our model, neither treatment appeared to substantially reduce damage within the ischemic core—a region that is highly vulnerable and typically undergoes irreversible injury, particularly when the core overlaps with the motor cortex, a structure essential for behavioral outcomes.

By contrast, the most pronounced differences between treatments occurred in the peri-infarct region, where tissue retains partial viability and is more amenable to neuroprotective interventions. Consequently, the similar neuromotor recovery observed across treatments may be primarily driven by preservation of functionally relevant cortical circuits, even if the degree of protection in the penumbra differed. These observations suggest that *B. procumbens* and Edaravone may exert distinct mechanistic patterns of action despite producing comparable behavioral outcomes.

At the molecular level, the MCAO + Bp group exhibited robust activation of the Nrf2 pathway, as demonstrated by the increased expression of total Nrf2 and the elevated levels of Nrf2-pS40, indicating functional activation of this master regulator of the endogenous antioxidant response. Phosphorylation of Nrf2 likely promoted its dissociation from KEAP1, enabling subsequent nuclear translocation and binding to antioxidant response elements (AREs) within the promoters of target genes such as *HMOX1*, *SOD*, *CAT*, and *GSR*, all of which play central roles in cellular defense against oxidative stress [44,45]. This mechanism is widely recognized as a critical component of neuroprotection in models of ischemic brain injury [46,47]. Several phytochemicals commonly present in plant extracts—including flavonoids, phenolic compounds, and coumarins—have been reported to induce activation of this pathway [48,49,50]. In line with this evidence, our findings suggest that a similar modulatory effect may occur with the *B. procumbens* extract, contributing to the enhanced antioxidant response observed in our study.

Recent evidence indicates that polyphenols present in plant extracts can elicit dose-dependent biphasic responses, a phenomenon known as hormesis, in which low concentrations activate adaptive cellular resilience mechanisms whereas higher doses may become counterproductive [51,52,53]. Based on our results, we suggest that the effects observed with the low dose of *B. procumbens* (40 mg/kg) are congruent with a hormetic response, as this treatment markedly increased Nrf2 activation. This suggests that its polyphenolic metabolites may act not only as direct antioxidants but also as modulators of adaptive cell-survival pathways in our ischemia/reperfusion model.

Consistently, a significant reduction in 4-HNE—a highly reactive lipid peroxidation marker associated with neuroinflammation and neuronal death [35]—was observed in ischemic rats treated with the extract. This anti-lipid peroxidation effect was also evident in the Edaravone group; however, it occurred in the absence of Nrf2 activation, which aligns with the established mechanism of Edaravone as a direct free radical scavenger rather than an inducer of endogenous antioxidant pathways [54].

Another relevant finding concerns AQP4. This water channel plays a critical role in the formation and progression of post-ischemic cerebral edema [14], and its overexpression has been associated with poorer functional outcomes [11]. In our model, AQP4 was markedly overexpressed in the MCAO group, and this pattern persisted in the MCAO + Eda group, suggesting that, despite its free radical scavenging capacity, Edaravone does not regulate AQP4 expression. In contrast, in the MCAO + Bp group, *B. procumbens* significantly reduced AQP4 protein levels, approaching basal values (as observed in the Sham group), indicating that the extract helps mitigate cerebral edema.

Histologically, this reduction in AQP4 expression was accompanied by an attenuated edema response. Our findings are consistent with previous evidence showing that Nrf2 activation decreases the pathological overexpression of AQP4 during ischemia/reperfusion. Studies by Fan et al. [55] and Liu et al. [56] demonstrated that Nrf2 activation indirectly modulates cerebral edema by downregulating AQP4 expression, whereas the absence of Nrf2 exacerbated gliosis and increased AQP4 levels. Conversely, pharmacological activation of Nrf2 reversed these alterations, supporting the notion that Nrf2 can modulate AQP4 expression.

Consistent with the previous findings, a significant recovery of NeuN expression was observed in the MCAO + Bp group compared with the untreated MCAO group, surpassing even the effect of Edaravone. This result suggests that the *B. procumbens* extract promotes superior neuronal preservation and, consequently, greater structural integrity of brain tissue, as also reflected in the H&E staining.

Beyond serving as a neuronal marker, NeuN plays functional roles in the regulation of alternative splicing in ion channels such as *SCN1A* (sodium channel) and *CACNA1C* (calcium channel) [57], modulates specific isoforms of the NMDA receptor [58], and influences the expression of plasticity-related genes such as *Egr4* and *Arc* [59]. Although these roles have not yet been demonstrated in the ischemic context, the marked decrease in NeuN observed in the MCAO group may contribute to altered electrophysiological properties, increased susceptibility to excitotoxicity, and impaired synaptic plasticity.

Interestingly, the MCAO + Bp group showed greater recovery of cortical cytoarchitecture than the Edaravone-treated group. This difference suggests that the extract may regulate brain water balance more effectively during the acute phase of infarction, likely by reducing the pathological overexpression of AQP4 and thereby limiting edema-related structural disruption.

Another relevant marker potentially involved in cell survival is Akt, which showed increased levels in both its total and Akt-pS473 forms in the MCAO + Bp group. Although the functional consequences of this change were not directly evaluated in this study, previous research has demonstrated that Akt activation contributes to pro-survival and neuroprotective signaling pathways, as described by Mullonkal et al. [60]. In other experimental contexts, Akt has also been shown to indirectly modulate Nrf2 activation through interactions with KEAP1, thereby facilitating its release and nuclear translocation [61].

Building upon the molecular pattern observed, we further propose that Akt activation in the MCAO + Bp group may contribute to pro-survival signaling while simultaneously facilitating conditions that support Nrf2 activation and a more regulated expression of AQP4. Taken together, these observations support the hypothesis that *B. procumbens* may influence the Akt/Nrf2/AQP4 axis, a possibility that merits further investigation.

Although pro-apoptotic targets were not directly evaluated in this study, previous research has shown that Akt negatively regulates key proteins such as p53, BAD, caspase-9, and Forkhead transcription factors, and participates in the modulation of inflammation through NF-κB and IKK signaling during ischemia/reperfusion [60]. Overall, this interaction network suggests that the neuroprotective effects of the *B. procumbens* extract may arise from a multifactorial mechanism in which antioxidant, anti-inflammatory, anti-apoptotic, and neuronal viability pathways converge.

In previous studies by our group [26], we identified Naringenin (29.2%), Equol 7-O-glucuronide (19.3%), and Paeoniflorin (8.2%) as the main metabolites present in the *B. procumbens* extract, collectively representing 56.7% of the total chemical profile. These compounds have been associated with neuroprotective and antioxidant effects, which may contribute to the biological outcomes observed in the present study.

Naringenin, the most abundant metabolite, has been widely reported as neuroprotective in ischemia models, where it improves cognitive performance, reduces lipid peroxidation, and enhances the activity of antioxidant enzymes such as SOD and GPx [62]. Gaur et al. [63] further demonstrated that Naringenin restores GSH and CAT levels and reverses mitochondrial and histological alterations in vulnerable brain regions, including the cortex and hippocampus. Although these enzymatic activities were not directly assessed in our study, the reduction in 4-HNE levels and the activation of Nrf2 observed in the MCAO + Bp group suggest a probable functional upregulation of this antioxidant network, consistent with the documented effects of this compound.

Equol 7-O-glucuronide and Paeoniflorin have also demonstrated neuroprotective properties. The former has been shown to reduce infarct volume and MDA levels in murine models [40], while Paeoniflorin has been reported to modulate inflammatory pathways by inhibiting TNF-α, IL-1β, iNOS, and COX-2 [64], proteins closely associated with oxidative stress and partially regulated by Nrf2 [65]. These findings suggest that the effects of the *B. procumbens* extract extend beyond redox control and may also involve modulation of the inflammatory response.

In addition, the *B. procumbens* extract contains other bioactive metabolites—including Genistein, Apigenin, *p*-hydroxybenzoic acid, and *m*-hydroxybenzoic acid—whose specific roles in cerebral ischemic injury remain to be clarified. Nevertheless, multiple studies have reported that these compounds possess relevant antioxidant properties, either as direct free radical scavengers or as inducers of the Nrf2/ARE pathway, thereby promoting the expression of cytoprotective enzymes such as HMOX1, NQO1, SOD, and CAT. These actions have been associated with beneficial outcomes in various models of oxidative stress–related diseases, including neurodegenerative, cardiovascular, and metabolic disorders [66].

PASS Online analysis suggests a high probability that several metabolites of *B. procumbens* act synergistically as antioxidants, free radical scavengers, inhibitors of lipid peroxidation, and modulators of redox enzymes involved in ischemia/reperfusion-induced brain injury mechanisms, consistent with previous reports by Sadeghzadeh et al. [67] and Wang et al. [9].

Notably, 12 of the 16 metabolites exhibited a high predicted probability of inducing *HMOX1*, a key Nrf2 target gene involved in protection against oxidative and inflammatory stress. The functional relevance of this enzyme has been demonstrated by Lu et al. [68], who showed that HMOX1 overexpression improves neurological outcomes, reduces infarct volume, and protects against neuronal apoptosis. In our study, the activation of Nrf2 observed following *B. procumbens* treatment suggests modulation of its downstream targets, potentially including *HMOX1*.

Furthermore, the Nrf2-centered interactome analysis predicted a dense network of proteins strongly associated with key processes such as inflammation, apoptosis, and oxidative damage, in agreement with the findings of Wang et al. [9]. This multitarget interaction pattern suggests that metabolites derived from *B. procumbens* may represent promising candidates for further investigation, given their potential to modulate multiple pathways implicated in the pathophysiology of ischemic brain injury.

**Figure 7:**
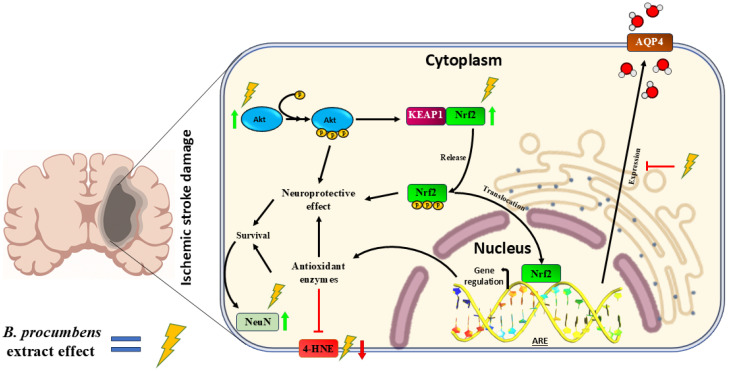
Proposed molecular mechanism exerted by *B. procumbens* metabolites in the MCAO model. Treatment with the aqueous extract appears to increase Akt and Nrf2 expression and activation, reduce lipid peroxidation, and decrease AQP4 overexpression, thereby contributing to the attenuation of cerebral edema. These effects converge to promote enhanced neuronal survival, as evidenced by increased NeuN expression, and collectively result in a neuroprotective response against ischemia/reperfusion-induced brain injury. Green arrows indicate increased protein expression, while red arrows indicate reduced expression after MCAO and *B. procumbens* treatment.

Figure 7 illustrates a proposed molecular mechanism by which *B. procumbens* extract may exert its neuroprotective effects in the ischemia/reperfusion model.

Ongoing in vivo experiments and in silico analyses are currently aimed at more precisely identifying the metabolites responsible for the observed effects. These studies include the evaluation of gene and protein markers associated with key pathways such as inflammation, apoptosis, and antioxidant defense—supported by interactome analyses—as well as the expression of Nrf2 downstream targets. The results will contribute to the selection of key bioactive compounds for developing more specific, safer, and highly promising therapeutic formulations.

## 4. Methods and Materials

### 4.1. Preparation of the Aqueous Extract of B. procumbens

The plant was cultivated under controlled greenhouse conditions. After collection, the plant material was air-dried at room temperature for 15 days and subsequently ground. Metabolite extraction was performed following the protocol previously described by Martínez-Cuazitl et al. [30].

Briefly, the dried material was pulverized and immersed in a hydroethanolic solution (water/ethanol, 1:1). The mixture was heated to 50 °C and stirred for 4 h. The solvent was then decanted and replaced with fresh extraction solution, and the procedure was repeated three additional times to maximize the recovery of bioactive compounds. Ethanol was removed by rotary evaporation, and the resulting aqueous fraction was lyophilized using a Telstar LyoQuest freeze-dryer (Telstar, Terrassa, Spain), yielding a dry brown powder suitable for experimental use.

### 4.2. Model of Induction of Transitory Focal Cerebral Ischemia in the Rat

Focal cerebral ischemia was induced using the middle cerebral artery occlusion model originally described by Longa et al. [69]. Briefly, male Wistar rats (280–350 g) were anesthetized with 2.5% isoflurane (PiSA, Guadalajara, Jalisco, México) delivered through a VerFlo traditional anesthesia system (Kent Scientific Corporation, Torrington, CT, USA), while maintaining 2% oxygen and a constant body temperature of 37 °C.

Cerebral blood flow to the middle cerebral artery was occluded by inserting a silicone-coated nylon filament (shaft diameter: 0.2 mm; tip diameter: 0.38–0.40 mm; RWD, MSRC40B200PK50) through the left internal carotid artery to a depth of 10 mm. Successful occlusion was verified by the immediate appearance of contralateral forelimb flexion following filament placement. After 2 h of occlusion, animals were re-anesthetized and the filament was gently withdrawn to allow reperfusion. Reperfusion proceeded for 48 h, after which the animals were euthanized.

Sham-operated controls underwent the same surgical procedure except for filament insertion. Physiological parameters were continuously monitored throughout the procedure, and animals were maintained at 37 °C during recovery until fully awakened from anesthesia.

### 4.3. Experimental Design

The animals were randomly assigned to four experimental groups (*n* = 6 per group):

(1) Sham, subjected to surgery without middle cerebral artery occlusion (MCAO).
(2) MCAO, undergoing surgery with middle cerebral artery occlusion (2 h of occlusion followed by reperfusion).
(3) MCAO + Bp, subjected to MCAO and treated with *B. procumbens* (40 mg/kg, intravenous).
(4) MCAO + Eda, subjected to MCAO and treated with Edaravone (0.45 mg/kg, intravenous).

Both treatments were administered via the tail vein immediately prior to reperfusion, with doses adjusted according to body weight. The aqueous extract of *B. procumbens* was dissolved in saline at a concentration of 40 mg/mL, whereas Edaravone was prepared at 1 mg/mL in saline containing 1% DMSO (*v*/*v*). All animals were euthanized 48 h after reperfusion.

Animal handling and experimental procedures were performed in accordance with the Declaration of Helsinki, the Mexican Official Norm for the care and use of laboratory animals (NOM-062-ZOO-1999) [70], and the guidelines for the disposal of biological hazardous waste (NOM-087-ECOL-SSA1-2002) [71]. The experimental protocol was approved by the Ethics Committee of the Postgraduate Division at ENMyH-IPN (approval number CBE009/2019).

The dose of *B. procumbens* used in this study was based on previous research from our group [30] and on reported data for structurally related compounds [72]. Likewise, the Edaravone dose was selected according to previously published studies evaluating its neuroprotective effects [73].

### 4.4. Neurological Outcome

Neurological function was evaluated 48 h after the ischemic insult and prior to euthanasia in all animals (*n* = 6 per group) using a battery of neuromotor tests commonly employed in rodent models of traumatic brain injury. The assessment included forelimb retraction, hindlimb extension, and lateral push resistance, each scored on a scale from 0 to 4. Animals were graded according to their performance in each task as follows: 4 = normal, 3 = mild deficit, 2 = moderate deficit, 1 = severe deficit, and 0 = afunctional, as previously described in studies assessing treatment effects [74]. A composite neuroscore was calculated for each animal by summing the individual test scores.

All behavioral assessments were performed by an investigator blinded to treatment allocation. In the forelimb retraction test, the rat is lifted by the base of the tail and gently placed back onto the surface to evaluate forepaw placement and posture. The hindlimb extension test involves lifting the rat by the tail after a backward slide, assessing hindlimb extension and toe spreading. The lateral push resistance test evaluates the animal’s coordination and resistance when the examiner attempts to rotate it into a supine position.

### 4.5. Measurement of the Injury Volume

Brains (*n* = 6 per group) were extracted and placed at −80 °C for 5 min. Coronal sections approximately 2 mm thick were then obtained and incubated in a 2% solution of TTC (Sigma-Aldrich, St. Louis, MO, USA) at 37 °C for 15 min. After staining, brain sections were photographed and analyzed using ImageJ software, version 1.54d (NIH, Bethesda, MD, USA).

To determine the proportion of infarcted tissue in the affected hemisphere, the percentage of infarct volume was calculated using the following formula:

$$\%\;of\;infarct\;Volume=\frac{[\sum \left(Injured\;areas\cdot thickness)](100\right)}{\begin{array}{c} Total\;area\cdot thickness\; \end{array}}$$

First, the volume of the left hemisphere in each slice was measured and multiplied by the corresponding slice thickness; the volumes were then summed to obtain the total volume of the affected hemisphere. Infarcted regions were subsequently identified and measured in each slice, and their respective volumes were summed to determine the total injured volume. This procedure allowed precise quantification of infarct extension relative to the total hemispheric volume.

### 4.6. Histopathological Analysis

After TTC staining, the slices (*n* = 3 per group) were washed in phosphate buffer and fixed in 4% paraformaldehyde at 4 °C for 24 h. The tissues were then processed for 16 h using a MICROM/STP120-1 tissue processor (Thermo Scientific, Walldorf, Germany) and embedded in Paraplast blocks (McCormick; Medex Supply, Brooklyn, NY, USA). Sections of 6 μm thickness were obtained from the blocks, stained with H&E, and subsequently evaluated for cortical morphology. Image acquisition was performed using an optical microscope (Olympus, Tokyo, Japan) equipped with a DP21 photographic system.

### 4.7. Western Blotting

Forty-eight hours after ischemia/reperfusion injury, protein expression was evaluated by Western blot (WB). Approximately 20 mg of cortical tissue (*n* = 3 per group) was collected and homogenized using an Ultra-Turrax T18 homogenizer (IKA, San Diego, CA, USA) at 5000 rpm for 1 min in RIPA buffer (150 mM NaCl, 1% Nonidet-P40, 1% sodium deoxycholate, 5 mM EDTA, 50 mM HEPES, pH 7.5) supplemented with protease inhibitors (Complete™, Merck, Darmstadt, HE, Germany).

Protein concentration was determined using the Bradford method [75]. Total protein lysates were first obtained independently from three rats per group, and after quantification, a pooled sample was prepared by combining 300 µg of protein from each animal (total: 900 µg). This strategy was employed due to the small size of the cortical tissue, allowing sufficient total protein to be obtained for the assays while minimizing interindividual variability. From this pooled extract, 30 µg of protein were loaded onto 12% polyacrylamide gels and subsequently transferred to polyvinylidene difluoride (PVDF) membranes (Merck, Darmstadt, HE, Germany) for immunodetection.

Membranes were blocked with 2% BSA prepared in PBS (pH 7.4) at room temperature and then incubated overnight at 4 °C with the following primary antibodies: anti-AQP4 (GTX133151), anti-NeuN (GTX30773), anti-4-HNE (GTX17571), anti-Nrf2 (GTX55732), anti–phospho-Nrf2 (Ser40) (GTX02873), anti-Akt (GTX121937), and anti–phospho-Akt (Ser473) (GTX128414) (all from GeneTex, Irvine, CA, USA), as well as anti–β-actin (sc-47778, Santa Cruz Biotechnology, Dallas, TX, USA). All primary antibodies were used at a 1:15,000 dilution.

Following primary antibody incubation, membranes were washed six times with PBS (pH 7.4) for 10 min each and then incubated with the appropriate secondary antibodies—anti-rabbit IgG (#111-035-003) or anti-mouse IgG (#115-035-062), both conjugated to horseradish peroxidase (HRP) (Jackson ImmunoResearch, West Grove, PA, USA)—depending on the host species of the primary antibody. After secondary incubation, membranes were again washed six times with PBS (pH 7.4) for 10 min prior to chemiluminescent detection.

Protein detection was performed using the Immobilon Western Chemiluminescent HRP Substrate kit (Sigma-Aldrich, Burlington, MA, USA). Chemiluminescent signals were visualized by exposing the membranes to Kodak autoradiography films, with exposure times adjusted (1–5 min) to ensure that band intensities remained within the linear detection range.

Densitometric analysis of protein bands was performed using ImageJ software, version 1.54d (NIH, Bethesda, MD, USA). β-actin served as the loading control for normalization. Although Western blot processing and densitometric quantification were not performed under blinding, all samples were analyzed using the same acquisition conditions and standardized ImageJ settings, which reduces the likelihood of analytical bias.

### 4.8. Prediction of the Antioxidant Activities of B. procumbens Metabolites

Based on previously identified metabolites in the aqueous fraction of *B. procumbens* [26], those with abundance values greater than 1% were selected. According to this criterion, a total of sixteen secondary metabolites were included. The corresponding SMILES codes for each compound were obtained from the PubChem platform (https://pubchem.ncbi.nlm.nih.gov/, accessed on 3 July 2024). As a comparative control, the compound Edaravone was also incorporated into the in silico analysis). As comparative control, the compound Edaravone was included in the in silico analysis.

Subsequently, to predict potential molecular targets or antioxidant-related activities, the SMILES codes of each metabolite were submitted to the PASS Online server, version 2.0, available on Way2Drug (https://www.way2drug.com/passonline/, accessed on 5 May 2025). Predictions were performed using the server’s default parameters, focusing exclusively on activities with probability values greater than 0.5, corresponding to a reasonable likelihood of biological activity according to the model’s training dataset [76]. Results were visualized as a heatmap using Microsoft Excel 365 (Microsoft Corporation, Redmond, WA, USA), employing a blue gradient scale to enhance interpretability.

### 4.9. Nrf2 Interactome Construction and Enrichment Analysis

To identify potential functional and regulatory interactions associated with Nrf2 in the context of its antioxidant role, an interactome was constructed using the STRING platform, version 12 (https://string-db.org/, accessed on 5 May 2025), selecting *Rattus norvegicus* as the reference species. The network was centered on the Nrf2 protein (NFE2L2), applying a minimum interaction confidence score of ≥70% and restricting the analysis to the top 100 proteins with the highest predicted interaction potential.

To complement the interactome analysis, functional enrichment was performed using two integrated tools within the STRING platform: Reactome pathway enrichment and gene–disease association enrichment (DISEASES).

### 4.10. Statistical Analysis

Graphs and statistical analyses were performed using GraphPad Prism, version 10.0 (GraphPad Software, San Diego, CA, USA). All data were obtained from a minimum of three independent experiments and are presented as mean ± SEM. Statistical comparisons were conducted using one-way or two-way ANOVA, followed by Tukey’s multiple comparison post hoc test to determine significant differences between groups. Statistical significance levels are reported according to the American Psychological Association (APA) format: * *p* ≤ 0.033, ** *p* ≤ 0.002, and *** *p* ≤ 0.001.

## 5. Conclusions

The extract of *B. procumbens* (40 mg/kg) improved neurological performance and reduced brain lesion size in a murine model of MCAO-induced ischemia/reperfusion injury. These effects were associated with decreased lipid peroxidation (4-HNE), activation of Nrf2—and likely the endogenous antioxidant system—reduced AQP4 expression, which may mitigate cerebral edema, and enhanced NeuN and Akt expression. Taken together, our findings provide preliminary evidence suggesting that *B. procumbens* may represent a promising therapeutic strategy for the treatment of ischemic brain injury.

## Data Availability

The original contributions presented in this study are included in the article. Further inquiries can be directed to the corresponding author.

## Abbreviations

The following abbreviations are used in this manuscript:

4-HNE	4-Hydroxy-2-nonenal
Nrf2	Nuclear factor erythroid 2–related factor 2
Nrf2-pS40 (phospho-Nrf2 Ser40)	Phosphorylated Nrf2 at serine 40
NeuN	Neuronal nuclei antigen/RNA binding fox-1 homolog 3
AQP4	Aquaporin-4
AKT	Protein kinase B (PKB/AKT)
Akt-pS473 (phospho-Akt Ser473)	Phosphorylated Akt at serine 473
β-catenin	Beta-catenin
KEAP1	Kelch-like ECH-associated protein 1
IL-1β	Interleukin-1 beta
IL-6	Interleukin-6
TNF-α	Tumor necrosis factor alpha
CASP3	Caspase-3
CASP9	Caspase-9
HMOX1	Heme oxygenase 1
HMOX2	Heme oxygenase 2
HIF-1α	Hypoxia-inducible factor 1 alpha
PARK7	Parkinsonism associated deglycase
JUN	Transcription factor Jun
FOXO (FOXO1/3/4)	Forkhead box O transcription factors
SIRT1	Sirtuin 1
NF-κB	Nuclear factor kappa-light-chain-enhancer of activated B cells
MAPK8 (JNK1)	Mitogen-activated protein kinase 8
p53 (TP53)	Tumor protein p53
NQO1	NAD(P)H quinone dehydrogenase 1
SOD1	Superoxide dismutase 1
SOD2	Superoxide dismutase 2
GSR	Glutathione reductase
TXN	Thioredoxin
GPXs (GPX1–8)	Glutathione peroxidases
